# Acceptance and Commitment Therapy and Psychological Well-Being: A Narrative Review

**DOI:** 10.7759/cureus.77705

**Published:** 2025-01-20

**Authors:** Anusuya S P, Gayatridevi S

**Affiliations:** 1 Women Studies, Avinashilingam Institute for Home Science and Higher Education for Women, Coimbatore, IND; 2 Psychiatry, Mary Lott Lyles Hospital, Madanapalle, IND; 3 Psychology, Avinashilingam Institute for Home Science and Higher Education for Women, Coimbatore, IND

**Keywords:** acceptance and commitment therapy (act), mindfulness, psychological flexibility (pf), psychological well-being (pwb), values-based action

## Abstract

Acceptance and commitment therapy (ACT) is an innovative psychological intervention emphasizing psychological flexibility and values-driven actions to enhance overall well-being. Unlike traditional cognitive-behavioral therapies (CBT), which often focus on altering maladaptive thoughts and behaviors, ACT encourages the acceptance of negative thoughts and emotions while fostering a commitment to personal values. This review explores ACT's effectiveness in promoting psychological well-being (PWB) by encompassing emotional, psychological, and social dimensions. Key aspects of PWB include emotional regulation, life satisfaction, and the reduction of experiential avoidance. The theoretical foundations of ACT are based on relational frame theory (RFT), which addresses cognitive fusion and experiential avoidance as central contributors to psychological distress. Extensive research, including meta-analyses and randomized controlled trials (RCTs), supports ACT's efficacy in treating various psychological issues and improving PWB. Despite strong empirical support, several gaps remain, including the need for more longitudinal studies, the exploration of cultural adaptability, and research on specific populations. Recommendations for future research include examining the long-term effects of ACT, its application across diverse cultural contexts, and its efficacy among underrepresented groups. This review underscores ACT's potential as a versatile therapeutic approach, providing valuable insights for mental health professionals and researchers.

## Introduction and background

Acceptance and commitment therapy (ACT) represents a modern and evidence-based psychological intervention aimed at enhancing psychological flexibility and overall well-being. Psychological flexibility, the cornerstone of ACT, refers to an individual's ability to remain open to their experiences, stay present at the moment, and engage in actions aligned with personal values, even when faced with emotional or cognitive discomfort [1]. This capacity is vital for maintaining mental health, enabling individuals to navigate life's challenges with resilience and purpose.

ACT was developed in the late 1980s by psychologist Steven C. Hayes and his colleagues as an evolution of behavior analysis and contextual behavioral science [2]. Rooted in the principles of relational frame theory (RFT), a behavioral account of human language and cognition, ACT diverged from traditional behavioral therapies by integrating acceptance and mindfulness techniques with behavior change strategies [3]. Hayes introduced ACT to address limitations he observed in existing cognitive and behavioral therapies, particularly their focus on symptom reduction rather than broader life enhancement [4].

Unlike traditional cognitive-behavioral therapies (CBT), which primarily aim to identify and restructure maladaptive thoughts, ACT emphasizes accepting these thoughts and emotions without judgment. Through mindfulness, individuals learn to observe their internal experiences with curiosity and openness rather than attempting to control or avoid them [5]. Simultaneously, ACT encourages commitment to actions guided by personal values, fostering a life of meaning and purpose. This unique combination of acceptance and values-driven behavior distinguishes ACT from other therapeutic modalities and provides a powerful framework for achieving psychological well-being (PWB).

The historical evolution of ACT reflects its foundation in both clinical practice and empirical research. Over the past three decades, ACT has been rigorously tested through randomized controlled trials (RCTs) and meta-analyses, demonstrating its efficacy across various psychological conditions, including anxiety, depression, and chronic pain [6,7]. The therapy's core principles are as follows: acceptance, mindfulness, cognitive defusion, values clarification, and committed action, a flexible approach that can be tailored to diverse populations and settings, making ACT a versatile and widely applicable therapeutic option [8].

As psychological therapies continue to evolve, there is a growing need to synthesize the extensive body of research on ACT to provide a comprehensive understanding of its mechanisms, applications, and long-term benefits. This narrative review aims to address this gap by analyzing current studies, exploring the unique aspects of ACT that contribute to psychological well-being, and discussing its implications for mental health professionals and researchers.

## Review

Overview of psychological well-being

Psychological well-being (PWB) is a multifaceted construct encompassing various dimensions of human functioning. It goes beyond the mere absence of mental disorders, including emotional, psychological, and social components that contribute to overall life satisfaction and quality of life. Understanding these components is essential for developing effective interventions such as ACT, which aims to enhance PWB [9].

Emotional Well-Being

Emotional well-being includes the experience of positive emotions and moods, such as happiness, joy, and contentment, as well as the absence of negative emotions such as sadness, anger, and anxiety. This dimension reflects an individual's general affective state and significantly contributes to overall life satisfaction [10]. Individuals with high emotional well-being tend to experience frequent positive emotions, which can buffer against stress and enhance overall mental health.

Psychological Functioning

Psychological functioning is characterized by self-acceptance, autonomy, environmental mastery, and personal growth. Self-acceptance refers to a positive attitude toward oneself and one's past experiences, acknowledging strengths and weaknesses. Autonomy involves the ability to make independent decisions and exercise self-determination. Environmental mastery denotes managing one's life and surroundings effectively, creating contexts that align with personal needs and values. Personal growth represents the continuous development and realization of one's potential, an ongoing process of self-improvement and adaptation to life's challenges [11].

Social Well-Being

Social well-being pertains to the quality of an individual's relationships and social roles. It encompasses feelings of belongingness, social integration, and the quality of interactions with others. Social well-being is crucial for creating a sense of community and connectedness, for overall well-being. Strong social connections are associated with numerous health benefits, including lower rates of morbidity and mortality, highlighting the importance of fostering positive social interactions [12].

Understanding PWB is critical as it significantly influences overall health, resilience, and quality of life. Individuals with high levels of PWB are better equipped to handle life's adversities and are more likely to experience positive health outcomes, including a reduced risk of chronic illnesses and greater longevity [13]. Additionally, PWB plays a vital role in promoting mental health, making it a key focus for therapeutic interventions such as ACT.

Theoretical foundations of ACT

ACT is rooted in relational frame theory (RFT), a comprehensive psychological framework that explores how human suffering often arises from the processes of language and cognition. RFT suggests that individuals form intricate networks of associations between words and concepts, leading to a phenomenon known as cognitive fusion. Cognitive fusion occurs when individuals perceive their thoughts as literal truths rather than recognizing them as transient mental events. For example, a person might internalize the thought "I am a failure" and treat it as an absolute reality [2]. This fusion can result in experiential avoidance, where individuals strive to evade or escape unpleasant thoughts, emotions, and sensations. Paradoxically, such avoidance can intensify psychological distress and hinder one's ability to engage fully in life [1].

ACT promotes six fundamental processes that collectively enhance psychological flexibility: acceptance, cognitive defusion, being present, self-as-context, values, and committed action. These processes are interrelated and work synergistically to help individuals lead a more meaningful and fulfilling life

Acceptance

Acceptance involves embracing all thoughts and emotions, including uncomfortable ones, as an inherent part of the human experience. This process encourages individuals to open up to their internal experiences rather than resisting or avoiding them, fostering a more adaptive and compassionate stance toward oneself [1].

Cognitive Defusion

Cognitive defusion refers to the creation of psychological distance from one's thoughts, helping individuals perceive thoughts as passing events rather than concrete truths. Cognitive defusion techniques, such as mindfulness and metaphor, aid in reducing the impact of unhelpful thoughts on behavior, allowing for greater flexibility in responding to various situations [1].

Being Present

Being present emphasizes maintaining a nonjudgmental awareness of the present moment, which can reduce rumination and alleviate anxiety. Mindfulness practices are central to this process, encouraging individuals to focus on their current experiences and engage fully with the present [1].

Self-as-Context

This approach involves understanding that one's identity is distinct from thoughts and feelings, fostering a stable sense of self amidst changing internal experiences. This perspective helps individuals maintain a consistent sense of self-identity, even as their thoughts and emotions fluctuate [1].

Values Clarification

This entails identifying what is truly important to an individual and aligning actions with these personal values. This process provides direction and purpose, helping individuals prioritize meaningful activities and pursue goals that align with their core beliefs [1].

Committed Action

Committed action encourages taking concrete steps toward goals that are consistent with one's values, promoting perseverance and adaptability. Committed action involves setting specific, values-driven goals and pursuing them despite obstacles, thereby enhancing overall life satisfaction and achievement [1].

These six core processes form the foundation of ACT and are essential for cultivating psychological flexibility. They provide a comprehensive framework for individuals to navigate life's challenges more effectively, fostering a more adaptive and fulfilling life [3].

Effectiveness of ACT

ACT's effectiveness has been extensively studied, with numerous randomized controlled trials (RCTs) and meta-analyses demonstrating its efficacy across various psychological conditions. A meta-analysis conducted by A-Tjak et al. (2015) reviewed multiple RCTs and found that ACT had moderate to large effects in reducing symptoms of anxiety, depression, and stress [6]. Recent trials highlight its impact in educational and workplace contexts, underscoring its role in improving adolescents' well-being and fostering workplace resilience [14-16]. Moreover, psychological flexibility has emerged as a key mechanism mediating stress and well-being. This evidence highlights ACT's utility in clinical settings and in promoting general well-being among non-clinical populations [16].

Another significant study by Fledderus et al. (2012) explored the impact of a self-help intervention based on ACT within a general population. The study revealed significant improvements in psychological flexibility, mindfulness, and life satisfaction. The participants reported a reduction in psychological distress, further underscoring ACT's potential to enhance PWB [5].

Additionally, a comparative study by Forman et al. (2007) evaluated ACT alongside traditional CBT for treating anxiety and depression. Both therapies effectively reduced symptoms, but ACT participants demonstrated greater acceptance and engagement in valued activities [17]. This suggests that ACT offers additional benefits beyond traditional therapies, particularly in promoting values-driven behavior and enhancing the overall quality of life.

Benefits of ACT for psychological well-being

Acceptance and commitment therapy (ACT) offers numerous benefits for psychological well-being (PWB), making it a valuable therapeutic approach for a wide range of individuals. Its core principles, acceptance, mindfulness, and commitment to values, are particularly effective in fostering mental health and enhancing the overall quality of life. Below are key ways in which ACT contributes to PWB.

Psychological Flexibility

One of the central goals of ACT is to cultivate psychological flexibility, which involves being open to a range of experiences and acting in ways consistent with one's values. This flexibility enables individuals to adapt to various life situations, reducing the impact of rigid thinking patterns and promoting overall mental health [9]. By fostering an open and accepting stance toward internal experiences, ACT helps individuals navigate life's challenges more effectively, leading to improved psychological well-being.

Emotional Regulation

ACT teaches acceptance and mindfulness techniques that help individuals manage their emotions more constructively. By acknowledging and accepting emotions rather than suppressing them, individuals can reduce the intensity and duration of negative emotional states. This process decreases distress and enhances emotional regulation, leading to a more balanced and fulfilling life [18]. The focus on emotional acceptance allows individuals to experience a broader range of emotions without becoming overwhelmed by them.

Life Satisfaction

A key aspect of ACT is aligning one's actions with personal values, which fosters a sense of purpose and direction. This alignment enhances life satisfaction by encouraging individuals to engage in meaningful activities and pursue goals that are truly important to them. The emphasis on values-driven behavior helps individuals find joy and accomplishment in their daily lives, even amidst challenges [5]. By focusing on what truly matters, individuals can achieve a greater sense of accomplishment and contentment. Studies integrating creative and artistic practices with ACT have shown promise in addressing grief and trauma, further broadening its applications [19].

Reduction of Experiential Avoidance

Experiential avoidance, or the tendency to evade unpleasant thoughts and feelings, is a common source of psychological distress. ACT directly addresses this issue by promoting acceptance of all experiences, including negative ones. This approach reduces avoidance behaviors that often exacerbate mental health issues, facilitating psychological growth and resilience [8]. By embracing a more open and accepting attitude toward uncomfortable experiences, individuals can reduce the negative impact of avoidance on their lives.

Long-Term Benefits

The benefits of ACT extend beyond immediate symptom relief. Research has shown that ACT's positive effects, such as increased psychological flexibility and improved emotional regulation, persist over time. These lasting benefits equip individuals with tools to navigate future psychological challenges, making ACT a valuable long-term investment in mental health [8]. The skills and strategies learned in ACT can be applied to various life situations, making it a versatile and enduring therapeutic approach [8].

Literature support for ACT and psychological well-being

Research supports the efficacy of ACT in enhancing psychological well-being. For instance, a meta-analysis by A-Tjak et al. (2015) found that ACT significantly reduces symptoms of anxiety, depression, and stress while also promoting life satisfaction and emotional regulation. This comprehensive review of the literature provides strong evidence for ACT's effectiveness across a wide range of psychological issues [6].

Further studies, such as those by Fledderus et al. (2012) [5] and Kashdan and Rottenberg (2010) [18], highlight ACT's role in enhancing psychological flexibility and adaptive emotional regulation strategies. These studies demonstrate that ACT reduces psychological symptoms and promotes positive mental health outcomes, making it a valuable therapeutic approach for individuals seeking to improve their well-being [5,18].

Clinical trials, such as those conducted by Forman et al. (2007), have confirmed ACT's effectiveness in treating depressive symptoms and encouraging engagement in valued activities. These findings collectively underscore the importance of ACT as a therapeutic approach for improving PWB and addressing a range of psychological issues. The evidence suggests that ACT's unique focus on acceptance, mindfulness, and values-driven living offers significant benefits for individuals experiencing a wide range of psychological challenges [17].

ACT's emphasis on acceptance, mindfulness, and values-based living makes it a crucial intervention for enhancing psychological well-being. By promoting psychological flexibility, emotional regulation, life satisfaction, and resilience, ACT provides individuals with essential skills for navigating life's complexities. Its evidence-based effectiveness and long-term benefits make it a highly recommended approach for those seeking to improve their mental health and overall quality of life.

Identified gaps in the literature

Despite the evidence supporting ACT, several gaps remain in the existing literature. First, there is a paucity of longitudinal studies examining the long-term effects of ACT on PWB. Understanding the durability of these effects over extended periods is crucial for determining the therapy's long-term efficacy. Such studies would provide valuable insights into how ACT's benefits persist over time and whether additional interventions are needed to maintain these effects.

Second, there is limited research on the application of ACT across diverse cultural contexts. Cultural differences can significantly influence psychological processes, making it essential to explore how ACT can be adapted to various cultural backgrounds. This exploration would help ensure that ACT is effective and culturally sensitive across different populations, addressing the unique needs and values of individuals from diverse cultural settings.

Third, more studies are needed to determine ACT's efficacy in specific populations, such as adolescents, older adults, and individuals with chronic illnesses. These groups may face unique psychological challenges that require tailored interventions. Research focusing on these populations would provide a more comprehensive understanding of ACT's applicability and effectiveness, helping to refine and adapt the therapy for different age groups and health conditions.

These identified gaps highlight the need for further research to expand the understanding of ACT's applicability and effectiveness. Addressing these gaps will provide a more comprehensive view of how ACT can be utilized across different populations and settings, ensuring that it remains a versatile and effective therapeutic approach.

Suggestions for future research

Future research should focus on conducting longitudinal studies to assess the long-term impact of ACT on PWB. Such studies would provide valuable insights into the sustainability of ACT's benefits and its effectiveness over extended periods. By tracking individuals' progress over time, researchers can better understand how ACT's core processes contribute to lasting improvements in psychological well-being.

Additionally, exploring the cultural adaptability of ACT is crucial, as cultural norms and values can affect therapy outcomes. Researchers should investigate how ACT can be modified to accommodate cultural differences, ensuring that the therapy is relevant and effective for individuals from diverse backgrounds. This research could involve collaborating with practitioners from different cultural contexts to develop culturally sensitive ACT interventions.

Researchers should also focus on underrepresented groups, including adolescents, older adults, and those with chronic conditions, to provide a more comprehensive understanding of ACT's applicability. These populations may have unique psychological needs that require specialized approaches. For example, adolescents may benefit from interventions that address developmental issues, while older adults may require support for age-related challenges. Research in these areas would help tailor ACT to meet the specific needs of these groups.

Finally, developing standardized measures to evaluate the outcomes of ACT interventions, including broader aspects of PWB such as life satisfaction and social functioning, would offer a more holistic evaluation of the therapy's effectiveness. These measures could help researchers and practitioners assess the full range of ACT's benefits, providing a more comprehensive understanding of how the therapy impacts individuals' lives.

By addressing these areas, future research can contribute to the refinement and expansion of ACT, ensuring that it remains a versatile and effective therapeutic approach across various contexts and populations. This research would help solidify ACT's place in the broader field of psychological therapies, providing evidence for its efficacy and adaptability.

Despite the evidence supporting ACT, gaps remain, particularly regarding its cultural adaptability and application in developmental transitions [20]. Future research should address these issues to maximize ACT's impact across diverse populations.

## Conclusions

Acceptance and commitment therapy offers a robust framework for enhancing psychological well-being through its unique focus on acceptance, mindfulness, and values-driven living. Empirical evidence supports its effectiveness across various psychological disorders and general mental health concerns. As ACT continues to evolve, its applications in diverse cultural and clinical settings are likely to expand, providing a valuable tool for promoting mental health and well-being.

By fostering psychological flexibility and encouraging values-driven actions, ACT helps individuals navigate life's challenges with greater resilience and satisfaction. The therapy's unique focus on acceptance and mindfulness distinguishes it from other therapeutic approaches, making it a valuable tool for enhancing PWB in clinical and non-clinical populations. As research continues to explore and refine ACT, its potential to improve the quality of life for individuals facing a wide range of psychological challenges will likely grow. Overall, ACT's emphasis on living in alignment with one's values and accepting all aspects of the human experience offers a holistic approach to mental health. Its evidence-based techniques and long-term benefits make it a highly recommended approach for those seeking to enhance their psychological well-being and overall quality of life. As the field of psychology advances, ACT is poised to remain a critical component of therapeutic practice, offering hope and healing to those in need.
